# Quantum super-oscillation of a single photon

**DOI:** 10.1038/lsa.2016.127

**Published:** 2016-08-26

**Authors:** Guang Hui Yuan, Stefano Vezzoli, Charles Altuzarra, Edward TF Rogers, Christophe Couteau, Cesare Soci, Nikolay I Zheludev

**Affiliations:** 1 The Photonics Institute, Centre for Disruptive Photonic Technologies, Nanyang Technological University, Singapore 637371, Singapore; 2 CINTRA, CNRS-NTU-Thales, CNRS UMI 3288, Singapore 637553, Singapore; 3 Optoelectronics Research Centre and Centre for Photonic Metamaterials, University of Southampton, Southampton SO17 1BJ, UK; 4 Institute for Life Sciences, University of Southampton, Southampton SO17 1BJ, UK; 5 Laboratory for Nanotechnology, Instrumentation and Optics, ICD CNRS UMR 6281, University of Technology of Troyes, Troyes 10000, France

**Keywords:** energy backflow, single-photon wavefunction, super-oscillation, super-resolution

## Abstract

Super-oscillation is a counterintuitive phenomenon describing localized fast variations of functions and fields that happen at frequencies higher than the highest Fourier component of their spectra. The physical implications of this effect have been studied in information theory and optics of classical fields, and have been used in super-resolution imaging. As a general phenomenon of wave dynamics, super-oscillations have also been predicted to exist in quantum wavefunctions. Here we report the experimental demonstration of super-oscillatory behavior of a single-quantum object, a photon. The super-oscillatory behavior is demonstrated by tight localization of the photon wavefunction after focusing with an appropriately designed slit mask to create an interference pattern with a sub-diffraction hotspot (~0.45 *λ*). Such quantum super-oscillation can be used for low-intensity far-field super-resolution imaging techniques even down to single-photon counting regime, which would be of interest to quantum physics and non-invasive and label-free biological studies.

## Introduction

Super-oscillation, in its general form, is a mathematical phenomenon in which a band-limited function can oscillate much faster than its highest Fourier component over arbitrarily large intervals^1, 2, 3, 4, 5^. The super-oscillation idea seems counterintuitive as it gives the illusion that the Fourier components of the function exist outside the spectrum of the function^6, 7, 8^. The physical implications of super-oscillations have been studied extensively in various fields of research including signal processing^9^, optics and quantum physics. In optics, super-oscillations result from a delicate near-destructive interference and exhibit rapid phase variations (or singularities) and high local momentum in relatively low-intensity regions, which has been used to beat the optical diffraction limit and inspires far-field super-resolution^10, 11, 12, 13, 14, 15, 16, 17^. Optical super-oscillations have been theoretically studied in Bessel beams, random waves and speckle patterns^18, 19, 20^, and experimentally generated by binary masks^13, 14, 21, 22, 23^, spatial light modulators^16^, optical pupil filters^24^ and planar metasurfaces^25, 26^.

In quantum physics, phenomena relevant to super-oscillations have also been intensely discussed theoretically. For instance, Aharonov *et al.* found that, although the initial boundary conditions of a quantum mechanical system can be selected independently of the final boundary conditions, it turns out that the weak measurement of a quantum system can have expectation values much higher than the spectrum of the operator^27, 28^. To name a few examples that can be derived from this observation, superluminal local group velocities were identified in evanescent optical fields^29^ and Klein-Gordon and Dirac waves^30^; spin-hall effect of photons can cause significant spatial beam displacement even if a slight change of preselected polarization state is made^31^. However, no experimental demonstration of super-oscillatory quantum behavior of photons has yet been performed. Although super-oscillations were well studied in the classical regime, it has not yet been shown that the super-oscillation phenomenon indeed holds at the single-photon level experimentally, which is far from being intuitive, even though expected.

In this work, we report the experimental observation that the wavefunction of a single photon can super-oscillate, and the wavefunction itself in a certain region contains sub-diffraction features with length scale smaller than that can be constructed with the allowable wavevector eigenvalues. Generation of a super-oscillatory pattern by a binary mask is in some ways analogous to Young’s classical experiment on diffraction from two parallel slits, but with some advantages. The super-oscillatory pattern is formed by a precisely tailored interference of multiple beams of different intensities and phases. The observation of super-oscillation with a single photon would be a further proof of Bohr’s principle for multiple beam interference with non-equal beams. Here Bohr’s wave-particle duality (complementarity) makes it impossible to observe both the wave (interference) effects and to know which path (slit) the photon particle actually took.

**Figure 1:**
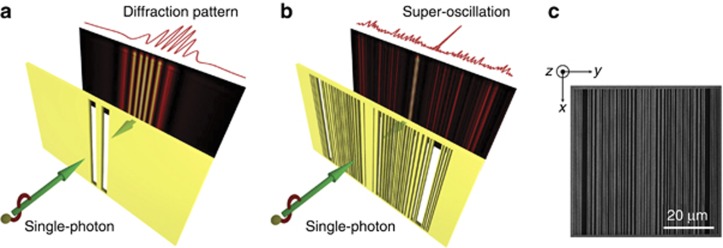
Single-photon regime of quantum interference. (**a**) Observation of quantum interference in the Young double-slit experiment. (**b**) Quantum super-oscillations with one-dimensional binary slit arrays (super-oscillatory lens). (**c**) Electron micrograph of the mask.

To demonstrate the super-oscillation behavior, we perform an experiment similar to the classical Young’s double-slit experiment shown in Figure 1a. Instead of the double-slit mask, we use a one-dimensional binary super-oscillatory lens (SOL) consisting of multiple parallel slits designed to construct classical super-oscillation interference pattern, as sketched in Figure 1b. This lens provides the availability for generation of a super-oscillatory state of a single photon. Similar to Young’s experiment that was performed with single photons, the wavefunction of a single photon passing through the slit array is a superposition of all possible paths and generates interference fringes. We expect that using the SOL, we generate a super-oscillatory interference pattern with a single photon. We investigate the properties of the field created by the SOL in terms of spatial confinement, local momentum and energy flow distribution.

## Materials and methods

### SOL design and fabrication

The SOL design is based on the powerful binary particle swarm optimization algorithm as we previously used^13, 14^. (See Supplementary Information section A for the design procedure.) The SOL is fabricated by focused ion beam (Helios 650, Hillsboro, OR, USA; 30 kV, 24 pA) milling through a 100-nm-thick gold film on an ITO glass substrate using a thermal evaporator (Oerlikon Univex 250, Cologne, Germany) with a deposition rate of 0.2 Å s^−1^. A 5-nm-thick chromium film was deposited in-between as an adhesion layer. The width deviation of the fabricated sample from the original design (integer multiples of unit size Δ*r*=400 nm) for each slit is <20 nm (~*λ*/40). Figure 1c shows a scanning electron micrograph of the SOL. It consists of 24 pairs of slits with different widths.

### Experimental setup for classical and single-photon measurements

**Figure 2:**
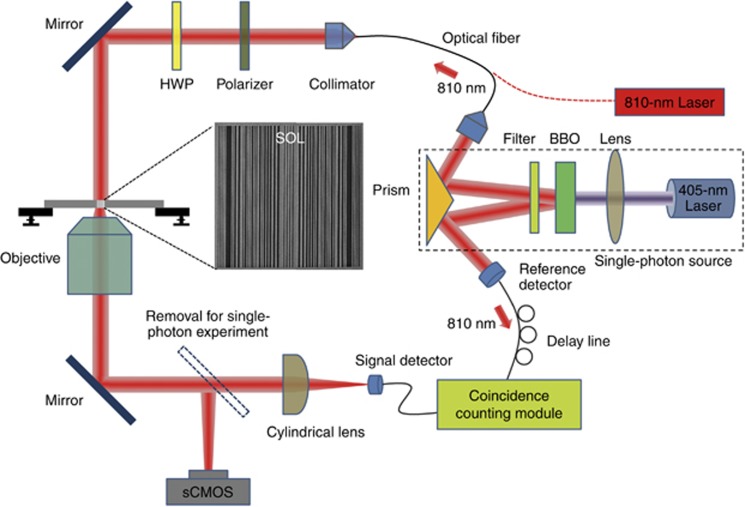
Experimental arrangements for observing single-photon quantum super-oscillations. The SOL is illuminated by a heralded single-photon source based on spontaneous parametric down-conversion in a BBO that is pumped by a 405-nm laser and produces correlated pairs of photons. One of the photons in the pair enters the SOL, whereas the other one is used as a trigger. The magnified field pattern created by the SOL is registered by scanning an optical fiber probe attached to a single-photon detector. To ensure single-photon regime of operation, the coincidence counts between the signal and reference channels are recorded. The same experiment arrangement is used for classical diffraction experiment with an external continuous laser operating at a wavelength of 810 nm, while diffraction pattern is recorded by a high-resolution sCMOS camera. BBO, beta-barium-borate crystal; HWP, half wave-plate.

Our experiment was performed both with a continuous laser and a source of heralded photons, and the schematic configuration is presented in Figure 2. For classical measurement, a linearly polarized continuous fiber laser source (Thorlabs MCLS1, Thorlabs Inc., Newton, NJ, USA; four-channel laser source) at wavelength of 810 nm is collimated and then illuminates the SOL from the substrate side. We used a high-magnification high-numerical aperture (NA) objective (Nikon CFI LU Plan APO EPI (Nikon Instruments Inc., Melville, NY, USA) 150 ×, NA=0.95) to collect the diffracted fields that are subsequently imaged by a high-resolution sCMOS camera (Andor Neo, Andor Technology Ltd., Belfast, UK; 2560 × 2160, pixel size 6.5 μm). Such magnification is essential due to the limited pixel size of the camera, but it will not undermine the super-oscillatory fields that are actually formed by interference of propagating waves, and thus can be mapped into the far-field and directly imaged by a conventional optical imaging system.

For the single-photon experiment, a 405-nm laser (LuxX 405-300 diode laser, Omicron-Laserage Laserprodukte GmbH, Rodgau, Germany) is used as the pump for producing a heralded single-photon source. After passing through the beta-barium-borate crystal at phase-matching angles, correlated photon pairs at the wavelength of 810 nm are generated via a spontaneous parametric down-conversion process^32^ and then split into two arms by using a prism mirror: one arm is directed onto the SOL with a beam diameter of ~70 μm and the other arm is coupled into an optical fiber and a delay line is used to count the coincidence of single photon. A cylindrical lens is used to focus the field in the *x* direction, and its long axis is precisely aligned to be perpendicular to the slit orientations so as to increase the intensity but not to change the field profiles in the parallel direction, and thus not distort the super-oscillatory fields. This is achieved by using a motorized precision rotation stage with angular resolution of 25 arcsec. In order to capture the field distributions in the propagation cross-section (*yz* plane), we use a single-axis piezo stage (Nanoflex/Thorlabs, Thorlabs Inc.) for *z* scanning of the SOL and for *y* scanning by a multi-mode fiber (MMF, aperture size ~62.5 μm) mounted on a long-range single-axis motorized translation stage (PT1/M-Z8/Thorlabs) and connected to a single-photon detector (Excelitas, Excelitas Technologies Corp., Waltham, MA, USA; dark count rate <250 cps). The scanning step sizes along *y* and *z* directions are 30 μm and 10 nm, respectively. The raster scanning is controlled by Labview programming and the integration time at each pixel is 3 s. The actual magnification factor in the *y* dimension is calibrated to be 306, which corresponds to an effective scanning step size of 98 nm.

Before carrying out the real single-photon experiment, we first attenuate the 810-nm CW laser, used in the classical measurement, down to the few photon regime using neutral density filters. This was done in order to precisely align the cylindrical lens and single-photon detector, and thus to maximize the transmission and collection efficiency of each optical element in the setup (see Supplementary Information section B for the estimation of overall efficiency). After the location of the hotspot is confirmed, we then switch to the heralded single-photon source to scan along the *y* direction, which is done by removing the flipping mirror in the optical path.

## Results and discussion

### Theoretically predicted and classically measured diffraction patterns

**Figure 3:**
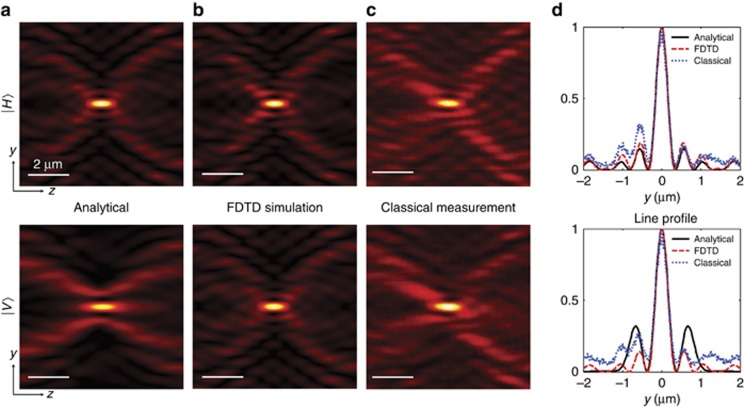
Calculated and measured classical super-oscillatory hotspots generated by SOL. The first and second rows represent data for incident light polarized along (|*H*〉) and perpendicular to (|*V*〉) the slits, respectively. (**a**) Vectorial angular spectrum method calculations; (**b**) FDTD simulation; (**c**) experimental maps; and (**d**) corresponding line profiles in the focal plane. In all cases, a laser at *λ*=810 nm was used. The field maps only show detectable transverse components of electric fields. FDTD, finite-difference time-domain technique.

We first compare our experimental results from classical laser interference with the theoretical diffraction patterns calculated by the vectorial angular spectrum method (Figure 3a; see Supplementary Information section C for details) and rigorous full-wave Maxwell simulation using finite-difference time-domain technique (Figure 3b). Here only the transverse electric fields are presented as the longitudinal component merely contributes to the transverse energy flow and is not registered in the experiment^33^. The two approaches agree very well, predicting a classical super-oscillatory hotspot with full-width at half-maximum (FWHM) of 0.4 *λ* at the distance *z*=10 μm away from the SOL. The energy concentration ratio inside the central hotspot is 7.5% and 9% for the parallel (|*H*〉) and perpendicular (|*V*〉) incident polarization, respectively (see Supplementary Information section D). For |*V*〉, the analytical model gives slightly more intense sidebands than numerical modeling, which should be attributed to the neglect of the multiple scattering in the analytical model.

The experimentally recorded diffraction patterns obtained in the classical regime by laser interference are shown in Figure 3c with corresponding line profiles plotted in Figure 3d. They match very closely to the theoretical predictions. The super-oscillatory peaks experimentally observed with |*H*〉 and |*V*〉 polarizations both have widths of 0.44 *λ*, which are undeniably smaller than the conventional diffraction limit determined by the availability of the highest harmonic in the spectrum: *λ*/(2NA_SOL_)=0.53 *λ*, where NA_SOL_=0.949 is the NA of the SOL mask with spatial extension of 60 μm and focal distance of 10 μm, by noticing that the super-oscillatory hotspots are generated by free-space Fourier component in the interval *k*∈[−NA_SOL_*k*_0_,NA_SOL_*k*_0_]. Moreover, our results are significantly smaller than an ideal diffraction-limited cylindrical lens with focal distance of 10 μm, giving spot sizes of 0.62*λ* and 0.7*λ* for |*H*〉 and |*V*〉 polarizations, respectively (see Supplementary Information section E).

Slight asymmetries of the experimental hotspot profiles and discrepancies between the theory and experiment are explainable by minor non-uniformity of the input laser wavefront, residual asymmetries of the structure due to fabrication tolerances, limited NA and spherical aberration of the imaging lens. In our experiment, the NA=0.95 of the objective lens imaging the diffraction patterns is comparable to the NA_SOL_ of the SOL mask, causing some clipping of the spectrum. This limitation of the imaging system becomes more noticeable when its object plane is closer to the SOL, which can be seen by comparing the left part of the first sideband near the hotspot in the experimental and theoretical diffraction patterns.

### Observation of single-photon super-oscillations

In the single-photon experiment, coincidence counts between the photons detected after the SOL and the ones collected in the second channel of the spontaneous parametric down-conversion process are used in order to ensure the presence of one and only one photon at a time. To confirm the single-photon character of the heralded source, we used a Hanbury Brown–Twiss setup to measure the second-order correlation function of the source. It was found to be *g*^(2)^(0)=0.088±0.029, that is, much smaller than 0.5, and thus is sufficient to claim essentially single-photon measurement regime^34^. (See Supplementary Information section F for measurement details of *g*^(2)^(0).)

**Figure 4:**
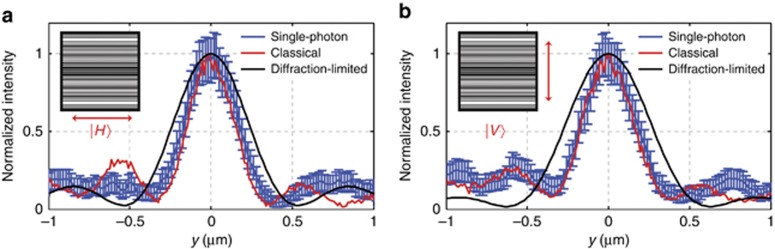
Super-oscillatory hotspot of a single photon. (**a**) |*H*〉 and (**b**) |*V*〉 polarizations. The error bars are defined as the square root of the observed coincidence counts. Classical measurement data show slightly smaller FWHM of the hotspot than that of single-photon measurement. The diffraction-limited hotspots given by an ideal cylindrical lens with the same focal length (10 μm) are also shown for intuitive comparison with the super-oscillatory focusing.

When measuring the super-oscillatory localization of the photon wavefunction, we scanned across the hotspot a number of times using a multi-mode optical fiber. In order to improve the signal-to-noise ratio, we averaged the data from 19 measurements. Figure 4 gives detailed measurement results. According to this, the super-oscillatory wavefunction of the single photon is confined in the hotspot with FWHM of (0.49±0.02) *λ* and (0.48±0.03) *λ* for |*H*〉 and |*V*〉 polarizations, respectively. This is slightly bigger than the calculated size of the hotspot (0.4 *λ*) and its measured value in the classical regime with a laser source (0.44* λ*). This raw result needs to be corrected by taking into account the instrumental limitations of the scanning setup using fiber probe of finite aperture with core size of 62.5 μm. The profile recorded by the detector is a convolution of the hotspot and the fiber aperture function, which increases the spot size by 6.8% (with account of magnification provided by the objective lens, see Supplementary Information section G for the details on the effect of finite fiber aperture to the hotspot size). Taking this into account, the super-oscillatory wavefunction of the single photon at the focus of SOL has a FWHM of (0.46±0.02) *λ* and (0.45±0.03) *λ* for |*H*〉 and |*V*〉 polarizations, respectively.

It is noted that, in principle, the size of the hotspot can be squeezed into arbitrarily small, but the detectable feature size is limited by realistic experimental conditions. With reducing hotspot size, the energy confined in the hotspot region decreases exponentially, and the weak-signal detection will be significantly constrained by the noise characteristic of the instruments, for example, the dark current in a CCD camera and the dark counts in a single-photon detector. Moreover, the limited pixel size of the detector also sets a trade-off between the achievable resolution and signal level: as magnifying the image of a super-oscillatory field or shrinking the aperture size of a detector (for example, fiber aperture of a single-photon detector), the collection efficiency will be decreased accordingly, which requires longer integration time to achieve a reasonable signal level, especially for the single-photon experiment.

Summarizing the experiment, we can conclude that we have observed super-oscillatory behavior of a single photon based on the following facts:

Within the experimental accuracy, the SOL generates hotspots of comparable size in both a classical interference experiment with coherent laser illumination and in the single-photon regime.
The hotspots generated by the SOL are demonstrably smaller than hotspot that could have been created by ideal cylindrical lens of the same size and focal distance.
The hotspots generated by the SOL are demonstrably smaller than the smallest hotspots that could have been created by highest values of the free-space wavevectors available as the result of scattering on the mask.

### Local wavevector and energy backflow near super-oscillatory hotspots

**Figure 5:**
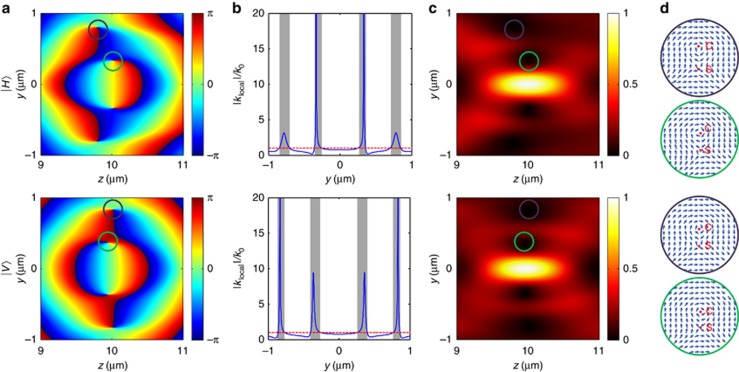
Phase, local wavevector and Poynting vectors near the super-oscillatory hotspots. The first and second rows represent data for incident light polarized along and perpendicular to the slits, respectively. (**a**) Phase profiles where the area with singular points are highlighted by purple and green circles; (**b**) *k*_local_ at *z*=10 μm where the super-oscillatory regions are shaded in gray and the red-dashed lines define |*k*_local_|=*k*_0_; (**c**) amplitude of Poynting vectors 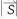. (**d**) Normalized Poynting vectors in purple and green circles clearly show the existence of center-type (C) and saddle-type (S) singular points and backward energy flow (negative *S*_*z*_).

Super-oscillation can be predicted either by observation of sub-diffraction localization or by the presence of high values of local wavevectors in the field distribution. To show that the SOL mask used in our experiment can indeed generate anomalies of the wavevector behavior, in Figure 5a, we plot the phase distributions *ψ* near the hotspots, where the phase of vector fields is presented according to the original definition from Pancharatnam and Berry^35^. The local wavevectors are then evaluated as the phase gradient *k*_local_=∇*ψ*. Lineouts at *z*=10 μm are shown in Figure 5b: there are several super-oscillatory regions where |*k*_local_|>*k*_0_, as marked with gray shading. There the phase oscillates up to 20 times faster than allowed by the maximum free-space Fourier component. The super-oscillation yield and maximized energy concentration ratio might be able to be optimized in a given spatial range of interest^36, 37^. A detailed evaluation of *k*_local_ along propagation direction can be found in Supplementary Information section H. In the phase map, we also observe several singular points of undefined local phase. The phase along a line encircling these points contains a complete phase cycle from ±*π* to ∓*π*. Such phase singularities often accompany super-oscillations, and squeeze the optical fields into a sub-wavelength scale (Figure 5c)^38^.

We also observed retro-propagation of the energy flow near super-oscillatory regions, as inferred from Figure 5d, that correspond to the enlarged area of highlighted purple and green circles in Figure 5a and 5c. For instance, the enlarged areas embraced by the purple and green circles contains center-type ‘C’ and saddle-type ‘S’ singular points. At these points, the magnetic field and electric field of the wave vanish, respectively, and in between them energy flows in the opposite direction to the incident wave. The ‘backflow’ of Poynting vector is accompanied by phase singularities that can be produced in any interference field and have been extensively studied in previous works^39, 40^. In quantum optics, such energy backflow in a super-oscillatory field is related to the negative eigenvalues of the local momentum quantum operator^41^. Although only theoretical proof is provided here, the direct measurement of the ‘backflow’ should be possible by measuring the associated photon momentum or wavevector using phase retrieval techniques in the framework of quantum weak measurement^42^.

## Conclusions

In summary, we have provided the experimental demonstration of quantum super-oscillations in the single-photon regime by using a specifically designed slit mask. High localization of the photon wavefunction with sub-diffraction hotspots of FWHM ~0.45 *λ* have been observed for both orthogonal polarization eigenstates better than the conventional diffraction limit of 0.53 *λ*. The degree of super-oscillation is modest, at only 15% less than the diffraction limit, due to the practical experimental conditions. There is a trade-off between the observed super-oscillatory hotspot size and single-photon counts. As the spot size is reduced further, the signal level of the super-oscillatory hotspot in the single-photon experiment will be getting close to the dark noise of the detector, which makes the experimental results not reliable. Our experiment is a proof of principle that a single photon can super-oscillate and it can beat the diffraction limit as well as ‘standard’ light. Meanwhile, the local momentum of the photon could surpass the expectation values restricted by the highest Fourier component of its band-limited spectrum. Our results illustrate that super-oscillations indeed result from the interference of a single-photon wavefunction with itself^43^, rather than from multi-photon interference. We anticipate our work to experimentally stimulate more fundamental studies in quantum physics, such as compression of photon’s wavefunction and energy ‘black-flow’ for quantum particles, and find various applications in quantum information such as quantum super-resolution imaging and quantum lithography, and also in non-invasive and label-free biological imaging^44^.

## Author contributions

GHY, ETFR and NIZ conceived the idea of single-photon super-oscillation. GHY and ETFR carried out the design and fabrication of the super-oscillatory lens. SV, GHY and CA performed the measurements and analyzed the data. CC analyzed the data and contributed to the experimental setup. GHY, SV and NIZ co-wrote the paper. NIZ supervised and coordinated all the works. All authors discussed the results and edited the manuscript extensively.
