# Supplementary material for: Quantum super-oscillation of a single photon
Source: Light Sci Appl. 2016 Aug 26;5(8):e16127–. doi: 10.1038/lsa.2016.127 (PMC6059939; doi:10.1038/lsa.2016.127)
Supplement: Supplementary information [file lsa2016127x1.docx]

Supplementary Information for

**Quantum super-oscillation of a single photon**

Guang Hui Yuan,^1^ Stefano Vezzoli,^1^ Charles Altuzarra,^1,2^ Edward TF Rogers,^3,4^ Christophe Couteau,^1,2,5^ Cesare Soci,^1^ and Nikolay I Zheludev^1,3*^

^1^TPI & Centre for Disruptive Photonic Technologies, Nanyang Technological University, Singapore 637371, Singapore

*^2^CINTRA, CNRS-NTU-Thales, CNRS UMI 3288, Singapore 637553, Singapore*

*^3^Optoelectronics Research Centre and Centre for Photonic Metamaterials, University of Southampton, Southampton SO17 1BJ, UK*

*^4^Institute for Life Sciences, University of Southampton, Southampton SO17 1BJ, UK*

*^5^Laboratory for Nanotechnology, Instrumentation and Optics, ICD CNRS UMR 6281, University of Technology of Troyes, Troyes 10000, France*

[^*^nzheludev@ntu.edu.sg](mailto:*nzheludev@ntu.edu.sg)

**Contents:**

1. Super-oscillatory lens design and optimization
2. Estimation of the optical efficiency of the system
3. Vectorial angular spectrum method to calculate the diffraction patterns and Poynting vectors
4. Energy concentration ratio inside the hotspots
5. Focusing performance of an ideal diffraction-limited cylindrical lens
6. Second-order correlation function measurement
7. Effect of limited aperture of single-photon detectors
8. Evolution dynamics of $k_{local}$ along propagation

**A. Super-oscillatory lens design and optimization**

The design of the super-oscillatory lens (SOL) is based on the powerful binary particle swarm optimization (BPSO) algorithm, which is a computational method that optimizes a problem with regard to a given merit function using a population of ‘particles’ in the *N*-dimensional search space.^1^ The *y* coordinate perpendicular to the slit (assumed to be orientated along *x*) is divided into *N*=75 pairs of slits, each of which has either unit or zero transmittance, and the BPSO algorithm searches for the best arrangement of opaque and transparent slits. The total mask size is 60 μm along both *x* and *y* directions and the slit width along *y* is ∆*r*=400 nm. The target function to describe the intensity profiles of electric fields near focus is defined as $exp(-\frac{y^{2}}{a^{2}}) exp\left[ -\frac{{(z-z_{0})}^{2}}{b^{2}} \right]$, where $z_{0}$ is the axial central position of the focal spot, $a=\frac{FWHM}{2\sqrt{ln2}}$, $b=\frac{DOF}{2\sqrt{ln2}}$, *FWHM* is the full-width half maximum of transverse spot size and *DOF* is the depth of focus. In the optimization, we used a swarm of 75 particles and 500 iterations. The optimum mask design is achieved at minimal variance between the actual intensity distribution and the merit function, as given in Figs. S1a,b respectively. The detailed parameters are given in Table S1. For an intuitive comparison, the original design and the scanning electron micrograph of the fabricated SOL are shown in Figs. S1c,d, where 24 pairs of slits with different widths are obtained.

| **Table S1.** Design parameters of the sample | | | |
| --- | --- | --- | --- |
| *z*_0_ (µm) | FWHM (λ) | DOF (µm) | Binary transmittance (starting from *y* center outward) |
| 10 | 0.4 | 1 | 010010000011001100110110110101100101011010101101000110011010111111010101010 |


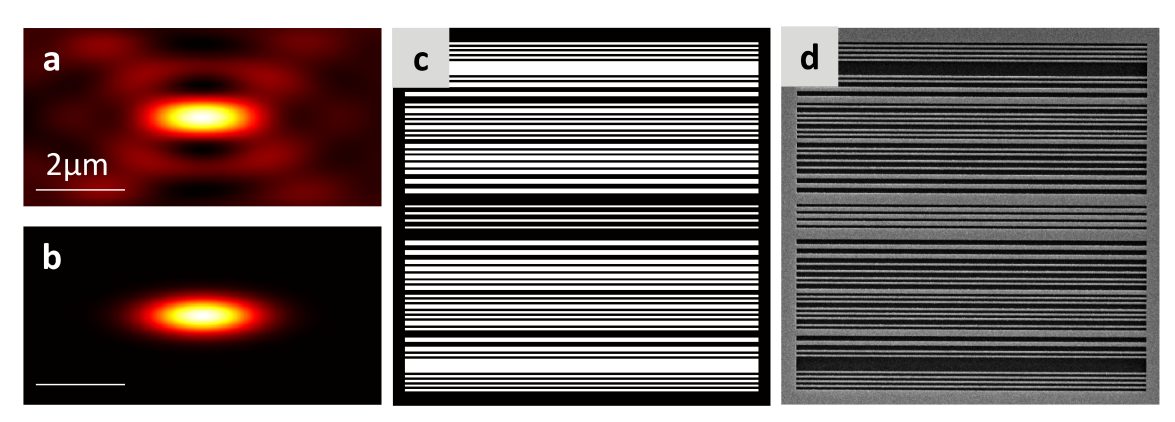


**Fig. S1.** (**a)** Actual intensity distribution of the optimized SOL (horizontal: 8 μm to 12 μm, vertical: -1 μm to 1 μm). (**b)** Defined merit function in the same region. (**c)** SOL design: the black and white regions denote zero and unit transmittance respectively. (**d)** SEM image of the fabricated SOL. The displayed areas are 64 μm by 64 μm.

**B. Estimation of the optical efficiency of the system**

We assess the losses and the coupling efficiency of the main optical elements in the setup for the sub-diffraction localization of the single photon, described in Fig. 2 of the main text.

In order to do so, we send into the setup a CW laser at 810 nm, as sketched in Fig. S2. The polarization optics can transmit up to *T*_pol_ = 70%, depending on the initial polarization state. The SOL sample has a transmission of *T*_sample_ = 16% when the beam diameter is set to around FWHM= 70 μm by use of a pair of confocal lenses. Finally the cylindrical lens allows for a coupling efficiency of *T*_sample_ = 30% into the 62.5 μm multi-mode fiber. The ratio between the intensity measured at the maximum of the main peak and the total intensity collected by a full scan along the *y* direction is *N*_max_ / *N*_tot_ = 3.4%.


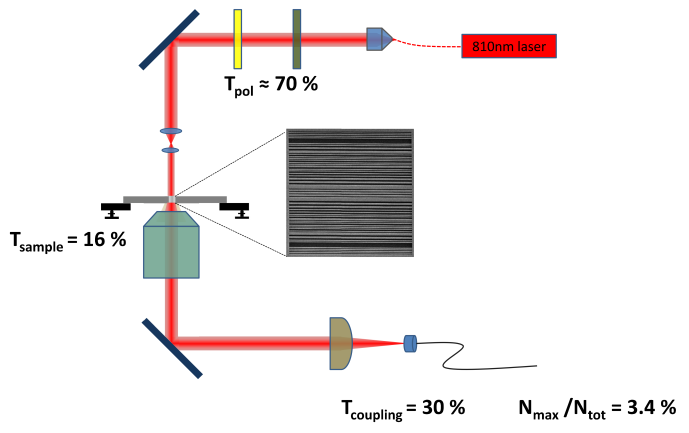


**Fig. S2.** Schematics of the setup for single photon sub-wavelength localization measurement, showing the losses and coupling efficiency of the main optical elements, measured with an 810 nm CW laser.

Starting with a rate of single counts of about *N* ≈ 100000 counts s^-1^ in the signal beam generated by down-conversion and coupled into a single mode fiber, we would expect *N* ≈ 120 counts s^-1^ at the maximum of the of the super-oscillatory peak, after the SOL and the coupling into the multi-mode fiber. We typically measure rates between 60 and 100 counts s^-1^. Corresponding coincidence counts are about 15-25 counts s^-1^, consistent with a detection efficiency of ~ 20% on the idler channel.

**C. Vectorial angular spectrum method to calculate the diffraction patterns and Poynting vectors**

The full electric field solutions of the Maxwell equation after passing through the SOL for the polarizations along ($\left. |H \right\rangle$) and perpendicular to ($\left. |V \right\rangle$) the slits can be respectively expressed as^2^

$\boldsymbol{E}_{\left. |H \right\rangle}\left( x,y,z \right)=E_{x,\left. |H \right\rangle}\left( x,y,z \right)\boldsymbol{e}_{x}+E_{y,\left. |H \right\rangle}\left( x,y,z \right)\boldsymbol{e}_{y}$ (1a)

$\boldsymbol{E}_{\left. |V \right\rangle}\left( x,y,z \right)=E_{x,\left. |V \right\rangle}\left( x,y,z \right)\boldsymbol{e}_{x}+E_{y,\left. |V \right\rangle}\left( x,y,z \right)\boldsymbol{e}_{y}+E_{z,\left. |V \right\rangle}\left( x,y,z \right)\boldsymbol{e}_{z}$ (1b)

where

$$E_{x,\left. |H \right\rangle}\left( x,y,z \right)=\iint\tilde{E}_{0}\sin^{2} \varphi\exp\left[ i\left( k_{t}\cos\varphi x+k_{t}\sin\varphi y+k_{z}z \right) \right]k_{t}dk_{t}d\varphi$$

$=\iint\tilde{E}_{0}\frac{k_{y}^{2}}{k_{t}^{2}}\exp\left[ i\left( k_{x}x+k_{y}y+k_{z}z \right) \right]dk_{x}dk_{y}$

$={FT}^{-1}\left\{ \tilde{E}_{0}\left( k_{x},k_{y},0 \right)\times\frac{k_{y}^{2}}{k_{t}^{2}}\times\exp\left( i\sqrt{k^{2}-k_{t}^{2}}z \right) \right\}$ (2a)

$E_{y,\left. |H \right\rangle}\left( x,y,z \right)=-\iint\tilde{E}_{0}\sin\varphi\cos\varphi\exp\left[ i\left( k_{t}\cos\varphi x+k_{t}\sin\varphi y+k_{z}z \right) \right]k_{t}dk_{t}d\varphi$

$=-\iint\tilde{E}_{0}\frac{k_{x}k_{y}}{k_{t}^{2}}\exp\left[ i\left( k_{x}x+k_{y}y+k_{z}z \right) \right]dk_{x}dk_{y}$

$=-{FT}^{-1}\left\{ \tilde{E}_{0}\left( k_{x},k_{y},0 \right)\times\frac{k_{x}k_{y}}{k_{t}^{2}}\times\exp\left( i\sqrt{k^{2}-k_{t}^{2}}z \right) \right\}$ (2b)

where $\tilde{E}_{0}\left( k_{x},k_{y},0 \right)=FT\left\{ E_{0}\left( x,y,0 \right) \right\}$ is the angular spectrum of the SOL located at *z*=0 , $FT$ and ${FT}^{-1}$ denote the Fourier transform and inverse Fourier transform respectively, $k_{t}=\sqrt{{k_{x}^{2}+k}_{y}^{2}}$ is the transverse wavevector, $\varphi$ is the polar angle, $k_{x}=k_{t}\cos\varphi$, $k_{y}=k_{t}\sin\varphi$, and $k_{z}=\sqrt{k^{2}-k_{t}^{2}}$.

The calculated electric field components at the focal plane (*z*=10 μm) are shown in Fig. S3, seen from which the $E_{y,\left. |H \right\rangle}$ component can be neglected since its amplitude is relatively small while $E_{x,\left. |H \right\rangle}$ is dominant and there are only slight distortions near the left/right edges of the horizontal slits.


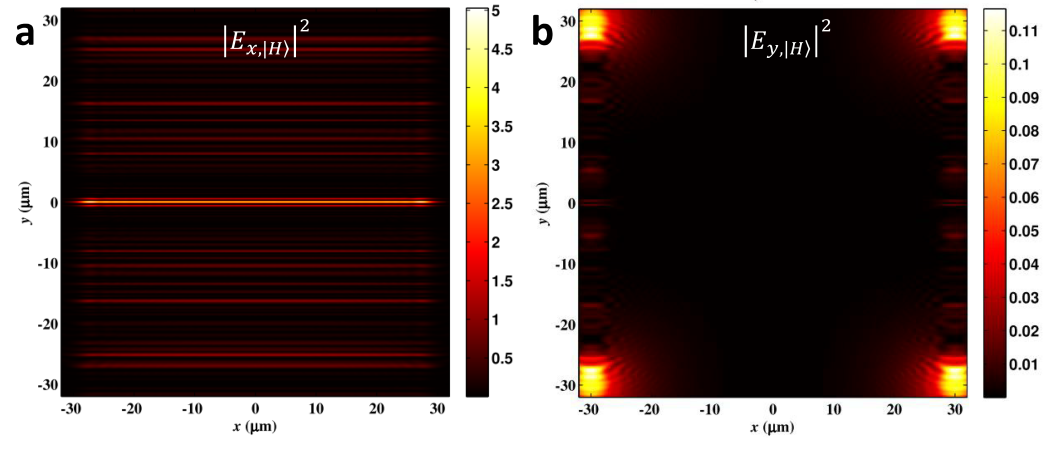


**Fig. S3.** Calculated electric field distributions at *z*=10 μm under $\left. |H \right\rangle$ excitation. (**a)** $E_{x,\left. |H \right\rangle}$ shows uniform intensity profile along the slit except at the left/right edges. (**b)** $E_{y,\left. |H \right\rangle}$ is small, inferred from the colorbar. There is no $E_{z}$ component for $\left. |H \right\rangle$ polarization.

In the similar way, for $\left. |V \right\rangle$ polarization,

$E_{y,\left. |V \right\rangle}\left( x,y,z \right)={FT}^{-1}\left\{ \tilde{E}_{0}\left( k_{x},k_{y},0 \right)\times\left( 1-\frac{k_{t}^{2}}{k^{2}} \right)\frac{k_{y}^{2}}{k_{t}^{2}}\times\exp\left( i\sqrt{k^{2}-k_{t}^{2}}z \right) \right\}$ (3a)

$E_{x,\left. |V \right\rangle}\left( x,y,z \right)={FT}^{-1}\left\{ \tilde{E}_{0}\left( k_{x},k_{y},0 \right)\times\left( 1-\frac{k_{t}^{2}}{k^{2}} \right)\frac{k_{x}k_{y}}{k_{t}^{2}}\times\exp\left( i\sqrt{k^{2}-k_{t}^{2}}z \right) \right\}$ (3b)

$E_{z,\left. |V \right\rangle}\left( x,y,z \right)={-FT}^{-1}\left\{ \tilde{E}_{0}\left( k_{x},k_{y},0 \right)\times\sqrt{1-\frac{k_{t}^{2}}{k^{2}}}\frac{k_{y}}{k}\times\exp\left( i\sqrt{k^{2}-k_{t}^{2}}z \right) \right\}$ (3c)

The Poynting vectors can thus be derived using the following expression

$\left\langle S \right\rangle=\frac{1}{2}\mathrm{Re} \left( E\times H^{*} \right)=\left\{ \begin{matrix} \frac{1}{2}\mathrm{Re} \left[ 0,-E_{x}H_{z}^{*},E_{x}H_{y}^{*} \right], \mathrm{for} \left. |H \right\rangle\\ \frac{1}{2}\mathrm{Re} \left[ 0,E_{z}H_{x}^{*},-E_{y}H_{x}^{*} \right], \mathrm{for} \left. |V \right\rangle\end{matrix} \right.$ (4)

where the asterisk denotes the complex conjugation.

The corresponding electric field components at the focal plane ( *z*=10 μm) are shown in Fig. S4. Similarly, the $E_{x,\left. |V \right\rangle}$ component is relatively small and negligible while $E_{y,\left. |V \right\rangle}$ and $E_{z,\left. |V \right\rangle}$ are dominant and only have slight distortions near the slit edges. $E_{z,\left. |V \right\rangle}$ appears owing to the projection of electric field into the axial direction under high-numerical-aperture focusing, and it has a double-peak and zero axial intensity due to destructive interference. However, this longitudinal component will not be detected by the CCD camera or single-photon detector due to polarization filtering.^3^


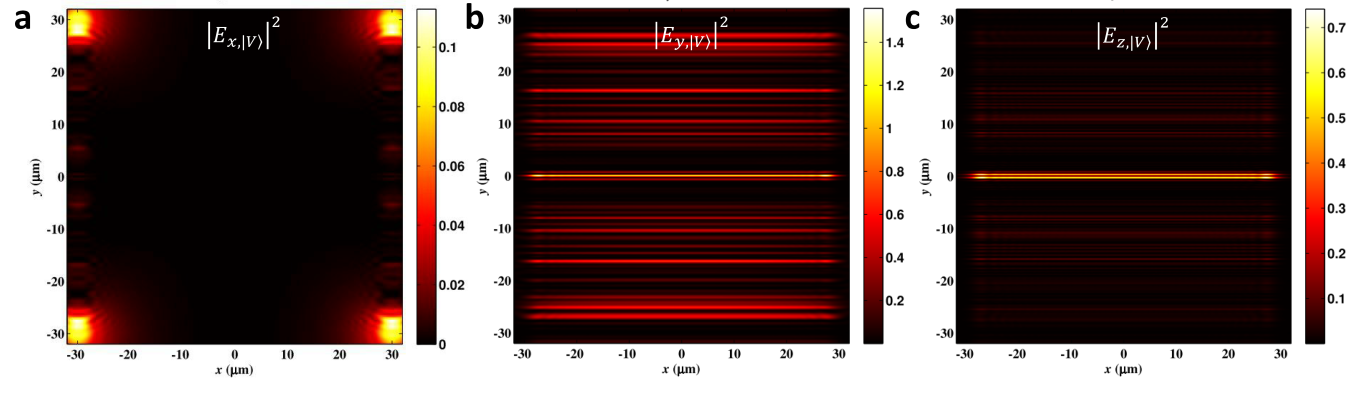


**Fig. S4.** Electric field distributions at $z=10 \mu m$ under $\left. |V \right\rangle$ polarization excitation. (**a)** $E_{x,\left. |V \right\rangle}$ is relatively small and can be neglected. (**b)** $E_{y,\left. |V \right\rangle}$ shows uniform intensity profile along the slits. (**c)** $E_{z,\left. |V \right\rangle}$ is null at *y*=0 due to destructive interference and has double-peak sidebands after high-NA focusing.

It is also worth noting that the plasmonic effect, that is the contribution from surface plasmon polaritons (SPPs), can also be neglected since the smallest slit width here is around half-wavelength which results in relatively low excitation efficiency of SPPs. This is confirmed by FDTD simulations where we use the perfect electrical conductor (PEC) boundary conditions, which do not support surface plasmons since electric fields cannot penetrate into them, to replace the real gold films and we obtain similar diffraction patterns shown in Fig. S5a,b for the case of $\left. |H \right\rangle$ polarization. From a line profile comparison at *z*=10 μm as shown in Fig. S5c, the two boundary conditions give the same peak positions and similar relative intensities of the hotspot and the sidebands. For $\left. |H \right\rangle$, surface plasmons cannot be generated due to momentum mismatching. We can conclude that surface plasmons will not dominate the far-field diffraction patterns in our experiment.


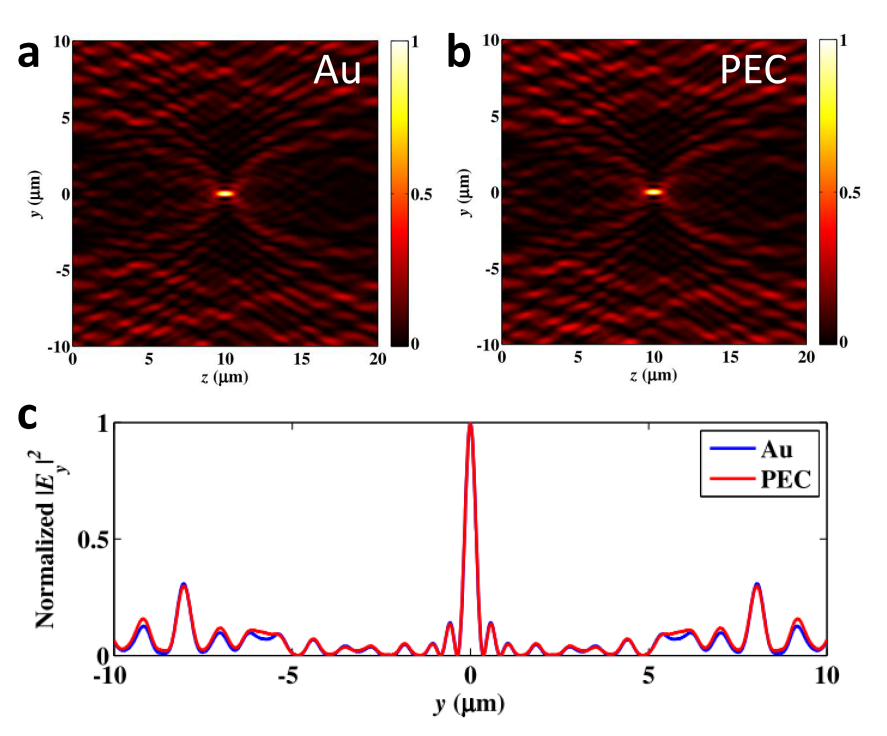


**Fig. S5.** Electric field intensity profiles under $\left. |V \right\rangle$ polarization using: (**a)** gold film and (**b)** perfect electrical conductor as the mask material. (**c)** Direct line profile comparison between **a** and **b** at *z*=10 μm.

**D. Energy concentration ratio inside the hotspots**

Using rigorous FDTD simulation, we have calculated the optical power distributions at the focal plane (*z*=10 μm) for both $\left. |H \right\rangle$ and $\left. |V \right\rangle$ polarizations, as shown in the Fig. S6. The energy concentration ratio inside the central super-oscillatory hotspot is found to be 7.5% and 9% for $\left. |H \right\rangle$ and $\left. |V \right\rangle$ polarization respectively. As the size of the hotspot is reduced further, less energy will be concentrated in the hotspot. The energy that can be channeled into the super-oscillatory region decreases exponentially with the number of super-oscillations.


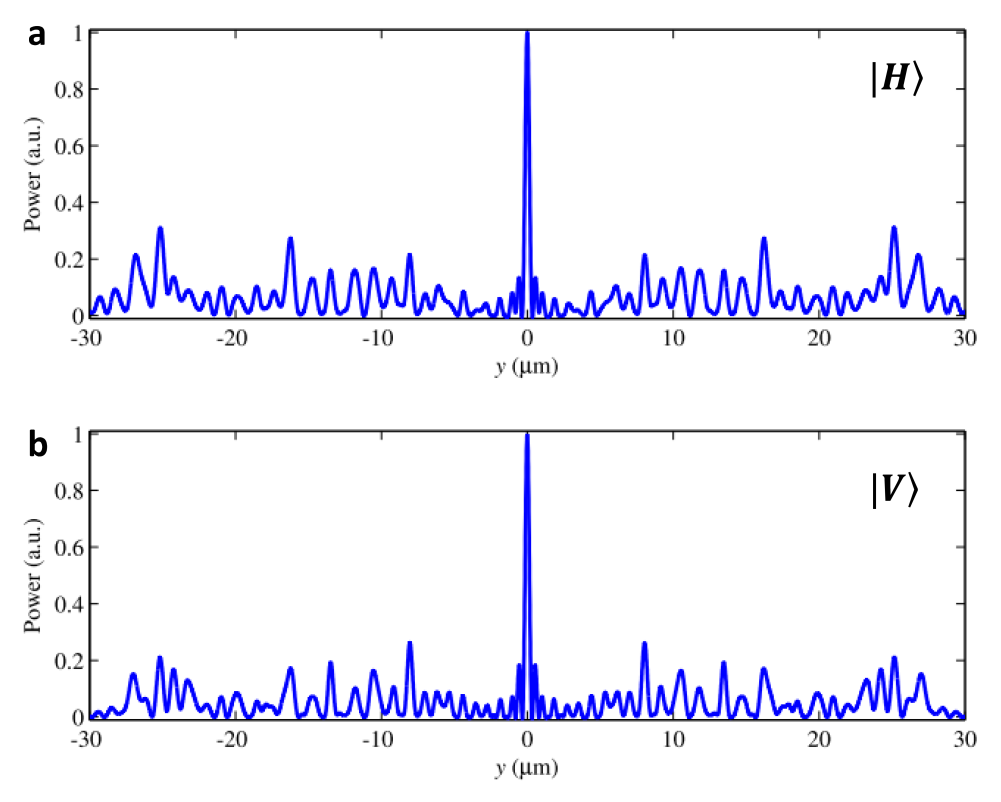


**Fig. S6.** Normalized optical power distributions at the focal plane: (**a)** $\left. |H \right\rangle$ and (**b)** $\left. |V \right\rangle$ polarization. The results are obtained from FDTD simulation.

For real applications, there are already several approaches to cope with the non-superoscillatory part of the signal (more specifically the sidebands). For example in super-resolution imaging: a) the hotspot generator can be designed and optimized to increase the intensity of the hotspot and lower the background sidebands. The peak electric field intensity of the hotspot $\left| E \right|^{2}$ in this work is 3 times higher than that of the sidebands. The intensity ratio between the highest sidebands and the hotspots is 0.26 and 0.31 for $\left. |H \right\rangle$ and $\left. |V \right\rangle$ polarizations respectively. This allows that most of the signals originate from the central hotspot, especially in nonlinear optical imaging where the signal would be possibly proportional to $\left| E \right|^{4}$; b) the field of view defined as the separation between the two nearest sidebands can be optimized and increased to an extent that only the signal coming from the hotspot will be recorded by a high-magnification objective in a confocal detection scheme which substantially reduces the scattering from the sidebands and diminishes the image distortion.

**E. Focusing performance of an ideal diffraction-limited cylindrical lens**


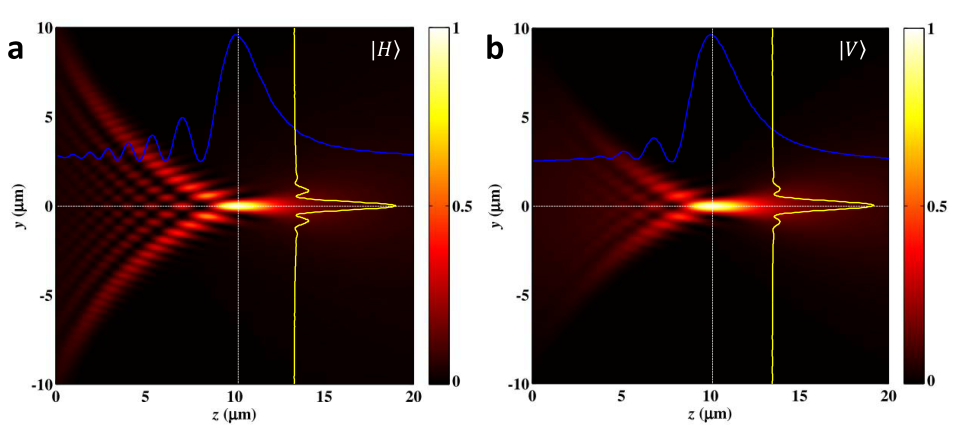


**Fig. S7.** Focusing properties of an ideal cylindrical lens with phase factor of $\exp\left( {-ik_{0}y^{2}}/f \right)$ which generates diffraction-limited hotspots with peak located at *z*=10 μm: (**a)** $\left. |H \right\rangle$ and (**b)** $\left. |V \right\rangle$ polarizations. The intensity profiles along the white lines in *y* and *z* direction are shown as yellow and blue curves respectively, where the value of $f$ is adjusted to be $f_{TE}$=11.9 μm and $f_{TM}$ =11.6 μm so as to achieve the focal length exactly as 10 μm. These patterns are calculated using vectorial angular spectrum method, showing hotspots with FWHM of 0.62λ and 0.7λ for $\left. |H \right\rangle$ and $\left. |V \right\rangle$ polarizations respectively.

**F. Second-order correlation function measurement**

We summarize the procedure for measuring the intensity auto-correlation function of the heralded single photon source, based on SPDC.

First of all, we recall the definition of the auto-correlation function for quantized fields where the intensity is measured at time *t*=0 and after a delay *t*= τ:

$g^{(2)}\left( \tau\right)=\frac{\left\langle\hat{E}^{-}(0)\hat{E}^{-}(\tau)\hat{E}^{+}(\tau)\hat{E}^{+}(0) \right\rangle}{\left\langle\hat{E}^{-}(0)\hat{E}^{+}(0) \right\rangle^{2}}$ (5)

For Fock states |n> (eigenstates of the intensity), the auto-correlation function at zero delay *g*^(2)^(0) can be easily calculated to be:

$g^{(2)}\left( 0 \right)=1-\frac{1}{n}$ (6)

Therefore, a measurement of *g*^(2)^(0) between 0 and 0.5 represents an experimental characterization of a true single photon source.

*g*^(2)^(τ) measurements are usually performed with a Hanbury Brown-Twiss (HBT) interferometer setup, as sketched in Fig. S8: the light is sent through a 50:50 beam splitter and the intensities in the two channels are measured by two detectors and electronically correlated. When single photon counters are used as detectors, the intensities are replaced by number of counts *N*_1,2_ and the product of intensities by the coincidence counts *N*_12_ detected within a window *Δ* (of typically few ns):

$g^{(2)}\left( 0 \right)=\frac{N_{12}}{N_{1}N_{2}}$ (7)

An SPDC source requires a heralded *g*^(2)^(0) measurement. Indeed, the non-linear down-conversion of a pump photon into two twin photons (called signal and idler) is a completely stochastic process. As illustrated in Fig. S8, the idler photon is used to gate the arrival of the signal on the other channel. Without the heralding’ of the photons, the statistics of the light arriving at the detectors would be that of a thermal field. In this case the auto-correlation function can be written as:

$g^{(2)}\left( 0 \right)=\frac{P_{12g}}{P_{1g}P_{2g}}$ (8)

Where *P*_ig_ is the conditional probability of detecting one photon on channel *i*, given the presence of a photon on channel *g*, for instance *P*_1g_ = *N*_1g_ /*N*_g ._ The final expression is:

$g^{(2)}\left( 0 \right)=\frac{N_{12g}N_{g}}{N_{1g}N_{2g}}$ (9)

thus requires the measurements of single count rate on the idler *N*_g_ (~5.23×10^6^ counts in a 20-second time window), coincidences between the idler and the signal on the two channels of the HBT setup *N*_1g_ (~5.1×10^4^) and *N*_2g_ (~2.1×10^4^), and triple coincidences *N*_12g_  (~18).


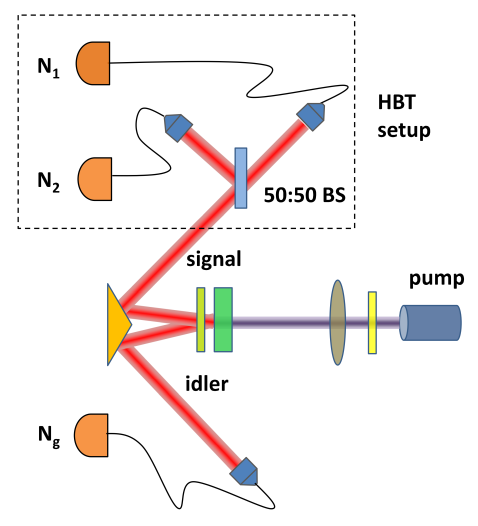


**Fig. S8.** Schematics of the setup for heralded *g*^(2)^(0) measurement. The counts on the idler *N*_g_ are used to herald the presence of photons in the signal beam inside the Hanbury Brown-Twiss (HBT) setup.

**G. Effect of limited aperture of single-photon detectors**

The effective pixel size of our single-photon detectors is 62.5 μm, which is given by the core size of the multimode fiber for collecting the photons. This large aperture size improves the collection efficiency but will limit the resolution. In fact, the signals we captured in the experiment are the convolution of the real signals (superoscillatory wavefunctions of the single photons) and aperture function of the multimode fiber. This will enlarge the super-oscillatory spot size. In our case, the 62.5 μm aperture shows an increment of 6.8% to the FWHM of the theoretically calculated hotspot while a 30 μm aperture would only give an increment of 1.6%, as shown in Fig. S9. This explains why the spot size in the single-photon experiment is larger than the theoretical prediction.


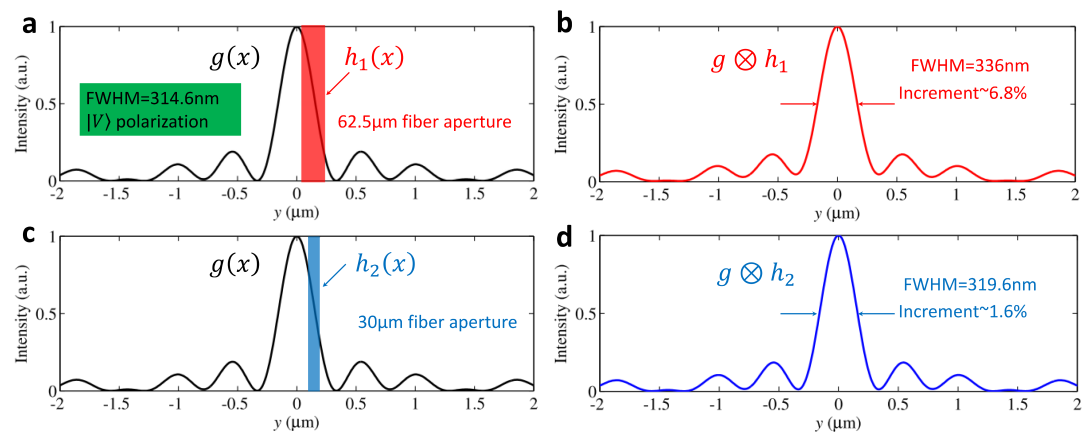


**Fig. S9.** Dependence of the measured super-oscillatory function $f\left( x \right)$on the aperture function of single-photon detector $h\left( x \right)$ and real super-oscillatory function $g\left( x \right)$: $f=g\otimes h$, $\otimes$ denotes the convolution. 62.5 μm and 30 μm fiber apertures increases the FWHM of the super-oscillatory spot by 6.8% and 1.6% as shown in (**a**,**b)** and (**c**,**d)** respectively. The FDTD simulated results under $\left. |H \right\rangle$ polarization are considered here for illustration. Note that in **a,b** the width of the drawn aperture has been reduced by a factor of 306, to take account of the magnification of the imaging system.

**H. Evolution dynamics of** $\boldsymbol{k}_{\boldsymbol{local}}$ **along propagation**

Super-oscillations can persist along propagation. The snapshots of the normalized local wavevectors along the +*z* direction ranging from 9.8 µm to 10.2 µm in steps of 0.1 µm are juxtaposed in Figs. S10a,b for $\left. |H \right\rangle$ and $\left. |V \right\rangle$ respectively. The peaks indicate the central positions of super-oscillations. $k_{local}$ becomes larger when getting closer to the points with phase singularities.


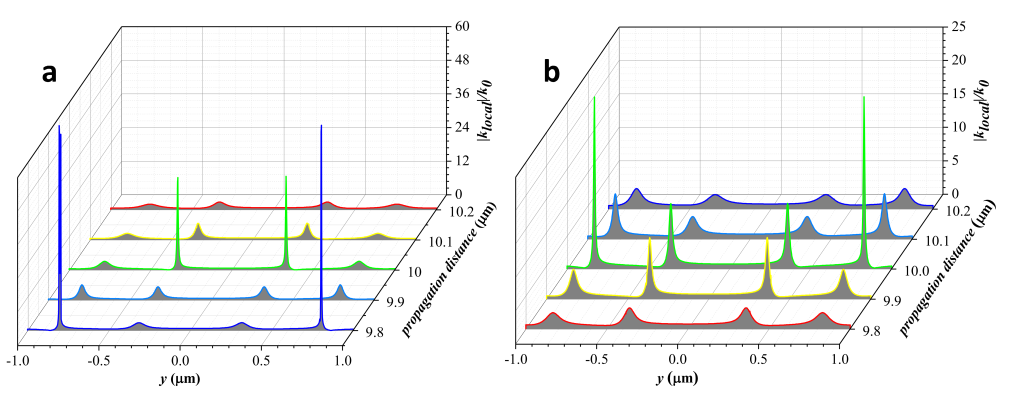


**Fig. S10.** $k_{local}$ evolution along propagation distance: (**a)** $\left. |H \right\rangle$ and (**b)** $\left. |V \right\rangle$ polarizations.

**References**

1. Jin N, Rahmat-Samii Y. Advances in particle swarm optimization for antenna designs: Real-number, binary, single-objective and multiobjective implementations. *IEEE Trans Antenn Propag* 2007; **55**: 556.
2. Martínez-Herrero R, Mejías MM, Bosch S, Carnicer A. Vectorial structure of nonparaxial electromagnetic beams. *J Opt Soc Am A* 2001; **18**: 1678.
3. Grosjean T, Courjon D. Polarization filtering induced by imaging system: effect on image structure. *Phys Rev E* 2003; **67**: 046611.
